# Quantification Supports Amyloid PET Visual Assessment of Challenging Cases: Results from the AMYPAD Diagnostic and Patient Management Study

**DOI:** 10.2967/jnumed.124.268119

**Published:** 2025-01

**Authors:** Lyduine E. Collij, Gérard N. Bischof, Daniele Altomare, Ilse Bader, Mark Battle, David Vállez García, Isadora Lopes Alves, Robin Wolz, Rossella Gismondi, Andrew Stephens, Zuzana Walker, Philip Scheltens, Agneta Nordberg, Juan Domingo Gispert, Alexander Drzezga, Andrés Perissinotti, Silvia Morbelli, Christopher Buckley, Valentina Garibotto, Giovanni B. Frisoni, Gill Farrar, Frederik Barkhof

**Affiliations:** 1 Department of Radiology and Nuclear Medicine, Amsterdam UMC, Vrije Universiteit, Amsterdam, The Netherlands;; 2 Brain Imaging, Amsterdam Neuroscience, Amsterdam, The Netherlands;; 3 Clinical Memory Research Unit, Department of Clinical Sciences Malmö, Faculty of Medicine, Lund University, Lund, Sweden;; 4 Department of Nuclear Medicine, Faculty of Medicine and University Hospital Cologne, University of Cologne, Cologne, Germany;; 5 Institute of Neuroscience and Medicine, Molecular Organization of the Brain, Forschungszentrum Jülich, Jülich, Germany;; 6 Department of Clinical and Experimental Sciences, University of Brescia, Brescia, Italy;; 7 Department of Neurology, Alzheimercenter, Amsterdam UMC, Vrije Universiteit, Amsterdam, The Netherlands;; 8 Neurodegeneration, Amsterdam Neuroscience, Amsterdam, The Netherlands;; 9 GE HealthCare, Amersham, United Kingdom;; 10 Brain Research Center, Amsterdam, The Netherlands;; 11 IXICO Plc, London, United Kingdom;; 12 Life Molecular Imaging GmbH, Berlin, Germany;; 13 Division of Psychiatry, Faculty of Brain Sciences, University College London, London, United Kingdom;; 14 LSP Life Sciences, Amsterdam, The Netherlands;; 15 Division of Clinical Geriatrics, Center for Alzheimer Research, Department of Neurobiology, Care Sciences, and Society, Karolinska Institutet, Stockholm, Sweden;; 16 Theme Inflammation and Aging, Karolinska University Hospital, Stockholm, Sweden;; 17 Barcelonaßeta Brain Research Center, Pasqual Maragall Foundation, Barcelona, Spain;; 18 IMIM, Barcelona, Spain;; 19 Universitat Pompeu Fabra, Barcelona, Spain;; 20 Biomedical Research Networking Center of Bioengineering, Biomaterials, and Nanomedicine, Carlos III Health Institute, Madrid, Spain;; 21 German Center for Neurodegenerative Diseases, Bonn–Cologne, Germany;; 22 Department of Nuclear Medicine, Hospital Clinic, August Pi i Sunyer Institute of Biomedical Research, Barcelona, Spain;; 23 Department of Medical Sciences, University of Turin, Turin, Italy;; 24 Nuclear Medicine Unit, Città della Salute e della Scienza di Torino, Turin, Italy;; 25 Division of Nuclear Medicine and Molecular Imaging, Geneva University Hospitals, Geneva, Switzerland;; 26 Department of Radiology and Medical Informatics, University of Geneva, Geneva, Switzerland;; 27 Center for Biomedical Imaging, Geneva, Switzerland;; 28 Laboratory of Neuroimaging of Aging, University of Geneva, Geneva, Switzerland;; 29 Geneva Memory Center, Geneva University Hospitals, Geneva, Switzerland; and; 30 Queen Square Institute of Neurology and Centre for Medical Image Computing, University College London, London, United Kingdom

**Keywords:** molecular imaging, neurology, visual read, Alzheimer, Centiloid quantification, amyloid PET

## Abstract

Several studies have demonstrated strong agreement between routine clinical visual assessment and quantification, suggesting that quantification approaches could support assessment by less experienced readers or in challenging cases. However, all studies to date have implemented a retrospective case collection, and challenging cases were generally underrepresented. **Methods:** We included all participants (*n* = 741) from the AMYPAD diagnostic and patient management study with available baseline amyloid PET quantification. Quantification was done with the PET-only AmyPype pipeline, providing global Centiloid and regional *z* scores. Visual assessment was performed by local readers for the entire cohort. From the total cohort, we selected a subsample of 85 cases for which the amyloid status based on the local reader’s visual assessment and the Centiloid classification (cutoff = 21) was discordant or that were assessed with low confidence (i.e., ≤3 on a 5-point scale) by the local reader. In addition, concordant negative (*n* = 8) and positive (*n* = 8) scans across tracers were selected. In this sample (*n* = 101 cases; [^18^F]flutemetamol, *n* = 48; [^18^F]florbetaben, *n* = 53), the visual assessments and corresponding confidence by 5 certified independent central readers were captured before and after disclosure of the quantification results. **Results:** For the whole AMYPAD diagnostic and patient management study cohort, overall assessment by local readers highly agreed with Centiloid status (κ = 0.85, 92.3% agreement). This was consistently observed within disease stages (subjective cognitive decline–plus, κ = 0.82, 92.3% agreement; mild cognitive impairment, κ = 0.80, 89.8% agreement; dementia, κ = 0.87, 94.6% agreement). Across all central reader assessments in the challenging subsample, quantification of global Centiloid and regional *z* scores was considered supportive of visual reads in 70.3% and 49.3% of assessments, respectively. After disclosure of the quantitative results, we observed improvement in concordance across the 5 readers (baseline κ = 0.65, 65.3% agreement; κ after disclosure = 0.74, 73.3% agreement) and a significant increase in reader confidence (baseline mean (*M*) = 4.0 vs. *M* after disclosure = 4.34, Wilcoxon statistic (*W*) = 101,056, *P* < 0.001). **Conclusion:** In this clinical study enriched for challenging amyloid PET cases, we demonstrate the value of quantification to support visual assessment. After disclosure, both interreader agreement and confidence showed significant improvement. These results are important considering the arrival of antiamyloid therapies, which used the Centiloid metric for trial inclusion and target engagement. Moreover, quantification could support determination of amyloid-β status with high certainty, an important factor for treatment initiation.

## Visual Abstract

**Figure:**
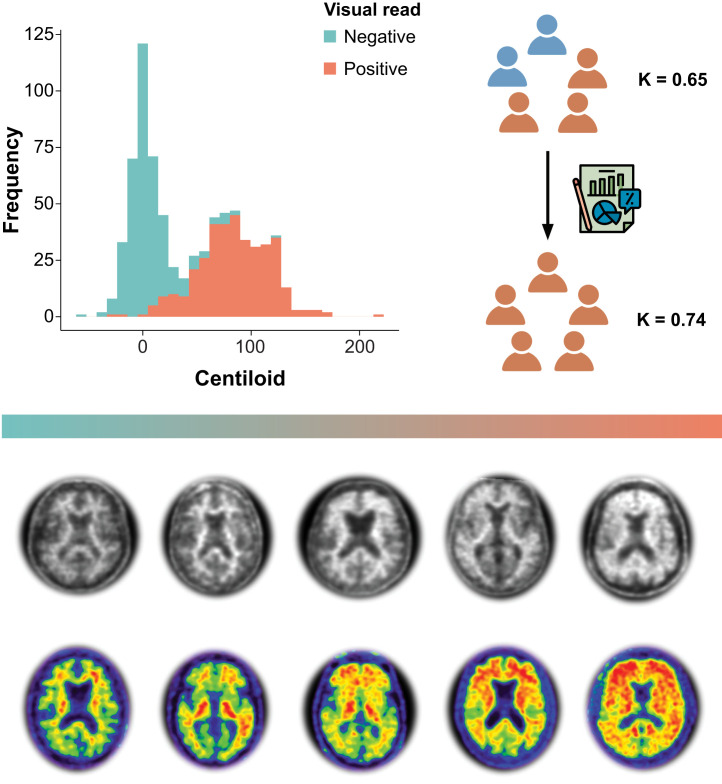

Recent advances in antiamyloid immunotherapies and their availability in routine clinical praxis makes it essential to determine the amyloid-β (Aβ) status of potentially eligible patients with high certainty (1). Within this context, quantifying Aβ PET for routine clinical use to support the diagnostic process of neurodegenerative disorders has received great interest in recent years (2). Several studies have demonstrated strong agreement between routine clinical visual assessment and quantification, suggesting that quantification approaches could support assessment by less experienced readers or in challenging cases (3–7). However, all studies to date have implemented a retrospective design, which did not allow direct assessment of the impact of quantification disclosure on visually based classification of Aβ status and of the confidence of the assessment. In addition, although most previous studies have speculated on the value of quantification to support particularly challenging cases, these are generally underrepresented; hence more detailed investigation is required to support this statement (6–8).

The 3 most comprehensive retrospective studies have illustrated strong agreement (86%–96%) between Aβ PET visual reads and several quantification approaches across the 3 ^18^F radiotracers approved by the U.S. Food and Drug Administration and European Medicines Agency (9–11). For [^18^F]flutemetamol, an average agreement of 94% between visual read and SUV ratio quantification derived from local nonharmonized quantification pipelines across 5 large clinical studies has been reported (6). A similar percentage agreement (i.e., 96.4%) has been reported for [^18^F]florbetaben, where visual read was compared with quantification across 15 software packages (7). Finally, in the arguably more real-world IDEAS dataset, consisting mostly of [^18^F]florbetapir scans, 86% concordance between visual read and Centiloid quantification using the robust PET-only processing pipeline has been demonstrated (12).

Centiloid quantification has been more widely implemented in recent years, because it brings the tracer-specific SUV ratio metric to a standardized scale, providing intuitive and across center/tracer generalizable cut points that reflect overall Aβ pathologic burden (13). Neuropathologic studies have shown that the earliest detectable Aβ PET signal occurs around 12 Centiloids, whereas 21–24 Centiloids best discriminates between subjects with no-to-low Aβ plaque burden and those with intermediate-to-high deposition (14*,*^15^) and 30 Centiloids is indicative of established Aβ burden (16). Compared with visual positivity, Centiloid cutoffs generally fall between these values, ranging roughly between 17 Centiloids for highly experienced readers (4) and 40 Centiloids in a routine clinical setting (17), although most consistently around 25 Centiloids (4*,*^5^*,*^8^*,*^12^*,*^14^). With consideration for the robustness of the measure (18), the Centiloid metric has been widely implemented in Alzheimer disease (AD) interventional trials. For example, lecanemab (Eisai) (19) and donanemab (Eli Lilly) (20) phase III trials have implemented Centiloids as their primary target engagement outcome and set a negativity threshold (Centiloids < 30 and 24.1, respectively) based on this quantification unit. Moreover, in the donanemab phase III trial, treatment was stopped if Centiloids were below 11 in a given scan or below 25 in 2 consecutive ones. Therefore, quantification could also be considered for the discontinuation of antiamyloid treatment in future clinical routine. It is thus key to familiarize routine clinical users with quantitative Aβ PET measures during their diagnostic work-up (5).

We aimed to determine the value of quantification in challenging clinical Aβ PET cases using a dataset covering the full diagnostic continuum. Here, we selected participants from the AMYPAD diagnostic and patient management study (DPMS) (21), who underwent Aβ PET imaging as part of their diagnostic work-up (22), and we assessed agreement between visual reads performed at each imaging site by local readers and quantification performed centrally. For the primary analysis, we selected a subset of challenging cases based on the local readers and assessed the agreement among 5 independent central readers before and after disclosure of quantitative results, as well as the confidence in their assessments.

## MATERIALS AND METHODS

### Cohort

Amyloid PET scans were obtained from the AMYPAD-DPMS randomized controlled trial (*n* = 840), which recruited patients across the disease continuum, including subjective cognitive decline–plus (SCD+), mild cognitive impairment (MCI), or dementia from 8 memory clinics across Europe. A detailed description of the baseline characteristics has been described previously (21). For the current work, the final disease stage (SCD+, MCI, or dementia) and etiologic diagnosis (AD, non-AD, or not yet achieved) during the DPMS observation period were used. The trial was registered with EudraCT (2017-002527-21). The study was approved by the Swiss institutional review board (Commission Cantonale d’Ethique de la Recherche) in Geneva (2017-01408), and all participants gave written informed consent.

### Patient Selection

All participants with an available baseline Aβ PET scan that passed quality control (described below) for quantification were included (*n* = 741). From this cohort, we selected a subsample of 85 Aβ PET scans for which the amyloid status based on local reader assessment and Centiloids (cutoff = 21, reflecting the lower level of Centiloids that best discriminates no-to-low and intermediate-to-high Aβ burden) (14) was discordant or that were assessed with low confidence (i.e., ≤3 on a 5-point scale; 1 = low, equivocal negative or positive; 5 = high, certain negative or positive) by the local reader (criterion 1, *n* = 38; criteria 1 and 2, *n* = 21; criterion 2, *n* = 26). In addition, concordant visual read and Centiloid negative (*n* = 8) and positive (*n* = 8) scans across tracers and sites to represent real-world negative and positive cases were selected from the total cohort to balance the dataset (*n* = 101; [^18^F]flutemetamol, *n* = 48; [^18^F]florbetaben, *n* = 53).

### PET Acquisition and Quantification

Scans were acquired according to the standard protocol for each tracer, that is, [^18^F]florbetaben (Neuraceq; Life Molecular Imaging) or [^18^F]flutemetamol (Vizamyl; GE HealthCare), starting 90 min after injection of 350 MBq (±20%) for [^18^F]florbetaben and 185 MBq (±10%) for [^18^F]flutemetamol and collected in 4 frames of 5 min each (90–110 min after injection). PET images were processed centrally using GE HealthCare’s AmyPype PET-only pipeline, providing global Centiloid (cortical target mask) and regional *z* scores (based on the AAL atlas; McGill University) for 6 cortical regions of interest (frontal, anterior cingulate, posterior cingulate, precuneus, lateral parietal, and lateral temporal cortex). AmyPype is an expansion of GE HealthCare’s conformité européenne-marketed CortexID software, which includes a reference population of 100 and 48 cognitively unimpaired healthy Aβ-negative controls who underwent [^18^F]flutemetamol or [^18^F]florbetaben, respectively, and is used to generate regional *z* scores. Amyloid PET images undergo frame-to-frame alignment and summing, and images are spatially normalized to the standard Montreal Neurologic Institute (MNI152) space using an adaptive template registration method (23). The whole cerebellum was used as the reference region. Agreement between AmyPype Centiloids and those obtained with the standard Centiloid pipeline has been previously established (24). For the primary analysis, Centiloids greater than 21 were considered the cutoff point for a positive Aβ PET scan. For illustrative purposes, scans were additionally classified as negative (Centiloids ≤ 10), intermediate or so-called gray zone (10 > Centiloids < 30), and positive (Centiloids ≥ 30).

Researchers interested in using the AmyPype software in their own research can request download instructions from amypype.downloads@gehealthcare.com.

### Visual Assessment

Visual assessment was performed according to established reader guidelines: by the local readers for the total cohort and by 5 certified independent central readers for the selected subsample. Images were rated, together with a T1-weighted MRI scan or CT scan, as either positive (binding in 1 or more cortical brain region unilaterally, or striatum in the case of [^18^F]flutemetamol) or negative (predominantly white matter uptake). In addition, regional classifications and reader confidence for both the final and the regional visual classifications based on a 5-point Likert scale were captured. To assess the effect of quantification disclosure on visual assessment by the 5 central readers of the subsample of challenging cases, visual read and corresponding confidence were captured before and after disclosure of the quantification results. Readers also stated whether Centiloid quantification or the regional *z* scores supported their assessment. Readers were masked to clinical information.

All subsample cohort readers received a short training session on the AmyPype processing pipeline, Centiloid quantification anchor points based on the review from Pemberton et al. (2), and *z* score quantification. The training material can be found in Supplemental Figures 1–4 (supplemental materials are available at http://jnm.snmjournals.org).

### Statistical Analysis

All analyses were performed in R Studio version 4.2.2 (R Project for Statistical Computing). Disease stage and etiologic diagnostic group differences in quantitative amyloid burden were assessed using ANOVA, corrected for age and sex. Agreement metrics were assessed using Cohen or Fleiss κ, when applicable. We performed bootstrapping (1,000-fold) on the Fleiss κ metric to retrieve the 95% CI of the metric and assess whether agreement showed a significant increase. First, agreement between local readers and Centiloid quantification status across the whole cohort and stratified by disease stage was assessed. Next, agreement between local readers and central readers was determined, where a majority visual read was based on the 5 readers (i.e., 3/5 assessments reflected majority Aβ status). Changes in reader confidence after disclosure of quantitative results were assessed using the Wilcoxon rank test, because the data were left-skewed.

## RESULTS

The total quantitative cohort consisted of 223 (30.1%) patients with SCD+, 258 (34.8%) patients with MCI, and 260 (35.1%) patients with dementia. The mean age was 70.8 ± 7.6 y, 44.8% were women, and the average Mini-Mental State Examination score was 25.5 ± 4.3. Overall, 49.5% of patients were considered visually Aβ PET positive based on local reader assessment (Table 1).

**TABLE 1.** Demographics

Parameter	SCD+			MCI			Dementia			Total cohort		
	AD	Non-AD	NYA	AD	Non-AD	NYA	AD	Non-AD	NYA	AD	Non-AD	NYA
*N*	37	105	81	119	95	44	194	49	17	350	249	142
Age (y)	70.2 ± 5.50	68.3 ± 6.21	68.7 ± 6.54	72.4 ± 6.73	70.0 ± 7.92	68.1 ± 10.5	73.1 ± 7.76	71.4 ± 7.96	70.5 ± 8.57	72.6 ± 7.25	69.6 ± 7.32	68.7 ± 8.15
Sex, F	15 (40.5%)	42 (40.0%)	39 (48.1%)	54 (45.4%)	36 (37.9%)	21 (47.7%)	102 (52.6%)	16 (32.7%)	7 (41.2%)	171 (48.9%)	94 (37.8%)	67 (47.2%)
MMSE	28.4 ± 1.71	28.6 ± 1.43	28.9 ± 1.48	25.9 ± 2.72	26.2 ± 3.42	26.5 ± 2.88	21.4 ± 4.53	24.2 ± 3.74	23.6 ± 4.13	23.7 ± 4.59	26.8 ± 3.28	27.6 ± 2.97
VR+	37 (100%)	13 (12.4%)	9 (11.1%)	106 (89.1%)	13 (13.7%)	5 (11.4%)	178 (91.8%)	3 (6.1%)	3 (17.6%)	321 (91.7%)	29 (11.6%)	17 (12.0%)
Centiloids	75.0 ± 34.1	9.85 ± 23.9	11.3 ± 28.3	75.9 ± 37.5	12.0 ± 30.8	10.3 ± 30.1	85.6 ± 38.7	8.47 ± 33.8	12.1 ± 34.3	81.2 ± 38.1	10.4 ± 28.7	11.1 ± 29.4

NYA = not yet achieved; MMSE = Mini-Mental State Examination; VR+ = visual read positivity.

Data are number followed by percentage in parentheses.

The challenging subsample included mostly MCI patients (52, 51.5%), followed by individuals with SCD+ (29, 28.7%), and finally dementia patients (20, 19.8%). The mean age of this subpopulation was 72.5 ± 7.6 y, 44.6% were women, and the average Mini-Mental State Examination score was 26.2 ± 3.7. Overall, 57.4% of patients were considered visually positive based on local reader assessment (Table 2).

**TABLE 2.** Demographics of Challenging Subsample Cohort

Parameter	SCD+	MCI	Dementia	Whole cohort
*n*	29	52	20	101
Age (y)	67.93 ± 5.7	73.87 ± 7.2	75.40 ± 5.9	72.47 ± 7.2
Sex, F	10 (34.5%)	26 (50.0%)	9 (45.0%)	45 (44.6%)
MMSE	28.6 ± 1.6	26.2 ± 3.2	22.9 ± 4.5	26.2 ± 3.7
VR+	14 (48.3%)	33 (63.5%)	11 (55.0%)	58 (57.4%)
Centiloid	32.8 ± 29.8	41.9 ± 31.8	27.1 ± 25.5	36.3 ± 30.4

MMSE = Mini-Mental State Examination; VR+ = visual read positivity.

Data are number followed by percentage in parentheses.

### Quantitative Amyloid Burden Across Diagnostic Groups

**FIGURE 1.**
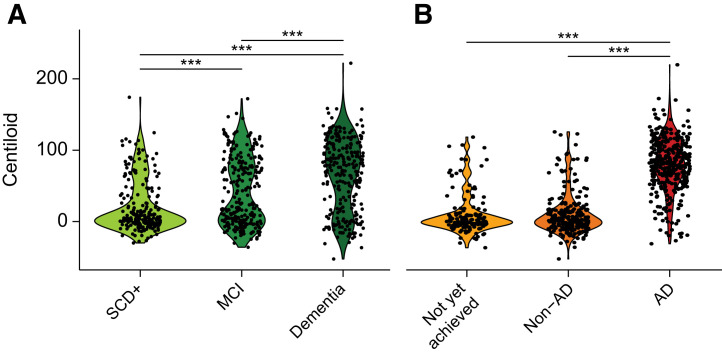
Centiloid quantification across disease stages and etiologic diagnosis. (A and B) Violin plot shows distribution of Centiloid burden across disease stages (A) and etiologic diagnosis (B). Significant differences between groups, after correction for age and sex, are illustrated. ***P < 0.001 is false discovery rate–detected.

Global amyloid burden expressed in Centiloids showed a stepwise increase with disease stage (SCD+ < MCI < dementia, *F* = 60.5, *P* < 0.001; Table 1; Fig. 1A) and was higher in AD than in the non-AD or not-yet-achieved etiologic diagnostic groups (*F* = 411.9, *P* < 0.001; Table 1; Fig. 1B). However, amyloid burden did not differ across the different clinical disease stages within etiologic groups (Supplemental Fig. 5). Regional *z* scores were highest in the AD group (all *P* < 0.01) but did not differ between the non-AD and the not-yet-achieved groups.

### Agreement Between Local Readers and Centiloid Quantification

**FIGURE 2.**
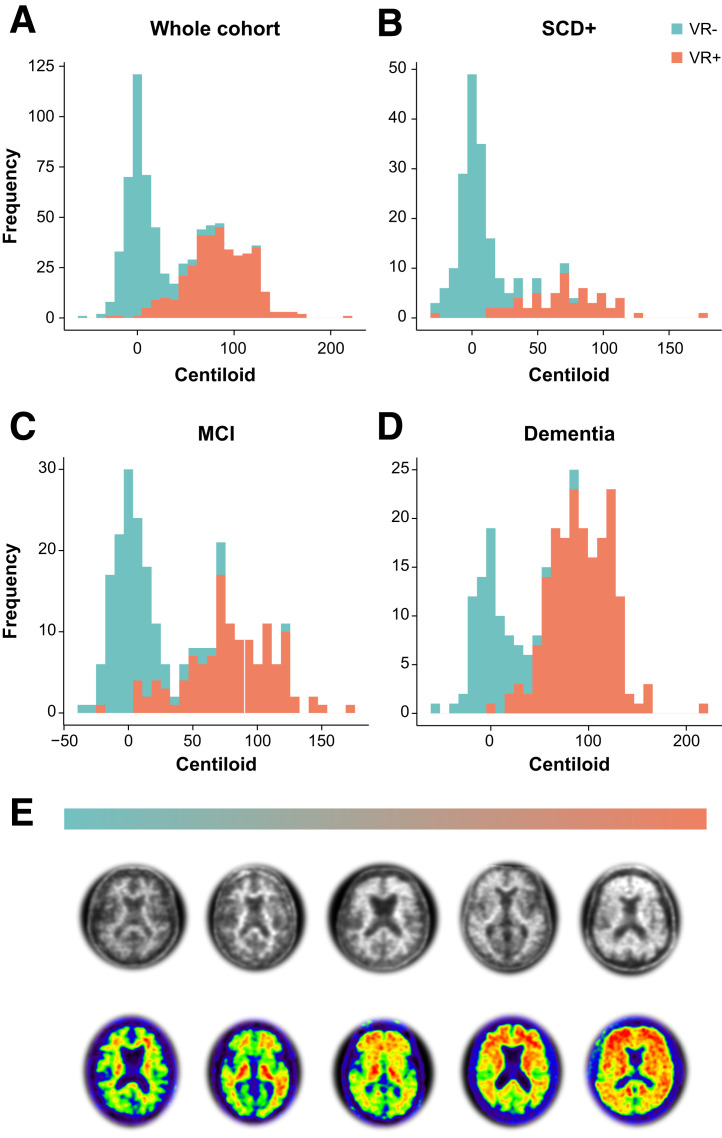
Agreement between local visual read and Centiloid quantification in whole DPMS cohort. (A–D) Histograms illustrate distribution of Centiloids across whole quantitative DPMS cohort (A), SCD+ patient population (B), MCI patient population (C), and dementia patient population (D). Bars are color-coded for visual read status by local assessor. (E) Illustrative Aβ PET from negative (left) to global positive (right). Top row represents [^18^F]florbetaben scans, and bottom row represents [^18^F]flutemetamol scans. VR = visual read.

For the whole quantitative cohort (*n* = 741), overall assessment by local readers highly agreed with Centiloid status based on the predefined cutoff of 21 (κ = 0.85, 92.3% concordance). This strong agreement was consistently observed within disease stage, ranging from κ equal to 0.87 (94.6% agreement) for dementia cases, κ equal to 0.82 (92.3% agreement) for SCD+ cases, and κ equal to 0.80 (89.8% agreement) for MCI cases (Fig. 2).

### Local Versus Central Readers

For the subsample enriched with challenging cases (*n* = 101), the agreement between local and central readers was, as per study design, low (κ = 0.21). With majority central read as the reference standard, local readers were more inclined to classify an Aβ PET scan as negative, resulting in 29 (28.7%) false-negative cases and 12 (11.9%) false-positive cases.

### Quantification Supports Visual Read of Challenging Cases

**FIGURE 3.**
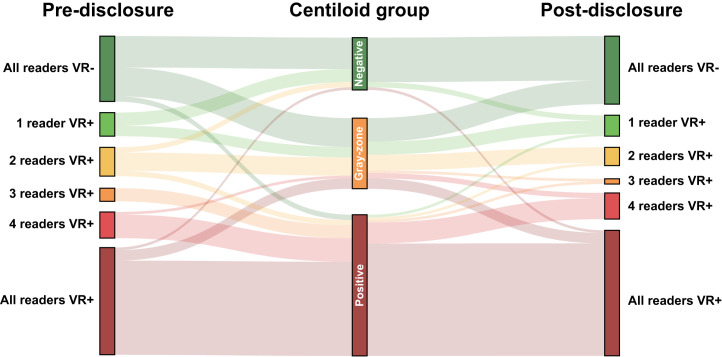
Change in visual read status after disclosure of quantitative results. Sankey plot illustrates changes in number of positive visual reads before and after disclosure of quantitative results. Change is illustrated by Centiloid group, that is, negative (Centiloids ≤ 10), gray zone (10 > Centiloids < 30), and positive (Centiloids ≥ 30). These Centiloid group classifications were not shared with reader but were created post hoc based on literature for visualization purposes. VR = visual read.

Across all 5 certified independent central reader assessments (*n* = 505), quantification of global Centiloid and regional *z* scores was considered supportive of visual assessment in 70.3% and 49.3% of assessments, respectively. Figure 3 illustrates changes in the number of positive visual assessments (0–5 as per number of readers) before and after disclosure of quantitative results. After disclosure of the quantitative results, we observed improvement in concordance across the 5 readers (κ before disclosure, 0.65; 95% CI, 0.55–0.74, 65.3% agreement; κ after disclosure, 0.74; 95% CI, 0.64–0.82, 73.3% agreement), which can be appreciated in the Figure 3 postdisclosure column, where relatively more cases were consistently visually negative or visually positive for all readers, although improvement in reader agreement did not reach statistical significance based on the bootstrapping-derived 95% CI of the κ metric. We also observed slight improvement in agreement between consensus read and amyloid status based on Centiloid (κ before disclosure, 0.53; κ after disclosure, 0.60). Finally, a significant increase in reader confidence after disclosure of quantitative results (*M* before disclosure, 4.0, vs. *M* after disclosure, 4.34; *W* = 101,056, *P* < 0.001) was observed.

**FIGURE 4.**
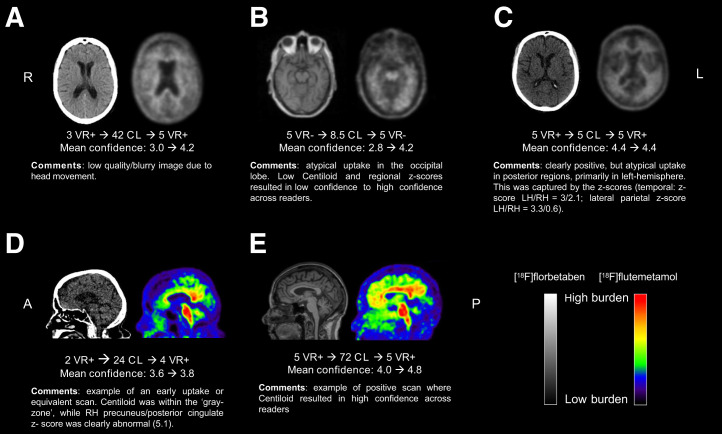
Examples of challenging cases. A = anterior orientation; CL = Centiloid; L or LH = left hemisphere; P = posterior orientation; R or RH = right hemisphere; VR = visual read.

Figure 3 further illustrates that reader agreement was particularly increased for cases with Centiloids greater than 30. Nonetheless, some cases did not reach consensus among readers or showed clear discrepancy between visual assessment and Centiloid quantification, particularly for those cases with a Centiloid value within the gray zone (Fig. 3). Examples are illustrated and further commented on in Figure 4.

### Possible Additional Value of Regional *z* Scores

As stated earlier, for 249 of 505 central assessments, the regional *z* scores were considered helpful in addition to the global Centiloid quantification. This was more apparent for 3 of 5 raters, who stated the regional *z* scores added to their read in 74, 67, and 62 of 101 cases, compared with 24 and 22 cases for the other 2 raters. For 48 cases across central readers, more detailed comments were provided, which suggested that the main benefit of regional *z* scores was in case of a borderline scan (28/48, 58.3%), particularly for the frontal and posterior cingulate or posterior cingulate cortex regions, followed by the quality of the image or atrophy (8/48, 16.7%). In 8 instances (16.7%), the regional *z* score caused confusion rather than further support of the initial visual assessment.

## DISCUSSION

In the prospective DPMS, we observed excellent agreement between local visual reads and Centiloid quantification across the clinical continuum. In a subgroup enriched for challenging cases, we demonstrate that improvement in reader agreement and confidence can be achieved using quantification results. Although overall agreement between local readers and quantification is strong and consistent across cognitive stages, approximately 11% of scans in the AMYPAD-DPMS cohort, representative of a typical memory clinical population, were considered challenging for various causes, such as suboptimal scan quality, atrophy, or borderline scan with emerging Aβ pathology. In the case of the latter, regional *z* scores might add support to the global Centiloid metric.

Our results suggest that quantification can support readers in determining Aβ status with high certainty, which is crucial considering the arrival of antiamyloid therapies, with their associated costs and potential side effects. Similar to previous studies (6*,*^12^*,*^25^), approximately 8% of AMYPAD-DPMS cases showed discordance between local readers and Centiloid quantification. Although the Centiloid cutoff used in the current work was at the lower end of the range that best discriminates no-to-low and intermediate-to-high Aβ burden (14), some considerable discrepancies were observed (Fig. 2). These examples possibly reflect misdiagnosis and could explain some previously reported discrepancies between visual read status and final diagnosis for this cohort (22). Even though the potential value of quantification has been previously demonstrated (3–7), most studies probably underestimated its true impact in a real-world scenario because of its limited value when visual assessment yields a clear positive or negative outcome. This study evaluated Aβ PET quantification performance in a range of amyloid loads from negative to positive (local reads) and in a subset of challenging cases in which quantification seems more beneficial, because visual analysis alone can be insufficient or less accurate. Nonetheless, quantification should always be done in conjunction with visual assessment to avoid misclassifications due to potential quantification errors. For example, 1 case had a low Centiloid value but was consistently assessed as visual read–positive by all readers (Figs. 3 and 4, case C).

In addition to high certainty in Aβ status, the extent of burden, as expressed in Centiloid units, has clinical relevance, considering the inclusion and discontinuation of treatment criteria implemented in 2 successful antiamyloid trials. More specifically, the lecanemab phase III trial defined amyloid positivity as a Centiloid value greater than 30, whereas donanemab used a Centiloid cutoff of greater than 37. The real-world IDEAS study demonstrated that around two thirds of the discordant cases were assessed as visually positive but classified as amyloid-negative based on Centiloid (12). In a future era of antiamyloid therapies, the adjunctive use of quantification could avoid such false-positive patients’ being unnecessarily medicated using regimens that potentially could last 1–2 y, have no therapeutic value, and have a risk of side effects. Although quantification is already added to the label of both radiotracers used in this study by the European Medicines Agency, current U.S. Food and Drug Administration guidelines for Aβ PET do not mention the added value that quantitation could bring to reaching high confidence and accurate determination of Aβ status based on visual reads. In addition, because the clearance rate for donanemab was so high, the study implemented a treatment discontinuation criterion, namely, when the Aβ PET quantification was a Centiloid value of less than 11 in a given scan or a Centiloid value of less than 25 in 2 consecutive scans. To what extent these specific cutoffs will be implemented in the user criteria for lecanemab and donanemab remains to be determined. For example, the current appropriate-use recommendations for lecanemab (26) elude only to a “positive Aβ PET or cerebrospinal fluid result indicative of AD.” Nonetheless, some initial results suggest a steady clearance rate of Aβ, independent of baseline amyloid burden (27). As such, future work should investigate whether the extent of baseline Aβ burden predicts necessary treatment duration to achieve full Aβ clearance. In such a setting, quantification will not only inform on Aβ status but also optimize individual treatment plans.

A limitation of the study is that we did not repeat visual assessments by local readers after disclosure of quantitative results. Although our central readers also had different levels of experience, this might have been ever more dispersed across the 11 onsite local readers. In addition, we had limited data to investigate whether subjects in the Centiloid gray zone would convert to Aβ-positive status at follow-up. Finally, in the current study, a Centiloid cutoff of greater than 21 to determine Aβ status was implemented to enrich the cohort with gray-zone cases, which is somewhat lower than found in previous studies and inclusion criteria for antiamyloid trials. Although central readers were masked to this information, and thus what constituted a Centiloid-based positive scan was up to the individual reader, this does raise the question of proper guidelines for the clinical use of Centiloid quantification. An opinion with regard to the clinical context of use of Centiloid quantification was recently adopted by the Committee for Medicinal Products for Human Use (EMADOC-1700519818-1200791) in a publication that states that a Centiloid threshold of greater than 30 is reflective of established amyloid pathology at the individual level with high certainty. This is in line with our findings, as illustrated in Figure 3.

## CONCLUSION

In this clinical study and subsample enriched for challenging Aβ PET cases, we demonstrate the value of quantification to support visual assessment. After disclosure, interreader agreement and confidence showed significant improvement. These results are important considering the arrival of antiamyloid therapies, which use the Centiloid metric for trial inclusion and target engagement. Moreover, quantification could support determination of Aβ status with high certainty, an important feature for treatment initiation.

## DISCLOSURE

The project leading to this paper has received funding from the Innovative Medicines Initiative 2 Joint Undertaking under grant agreement 115952. This joint undertaking receives support from the European Union’s Horizon 2020 research and innovation program and EFPIA. This communication reflects the views of the authors, and neither IMI nor the European Union or EFPIA is liable for any use that may be made of the information contained herein. Lyduine Collij has received research support from GE HealthCare and Springer Healthcare (funded by Eli Lilly), both paid to the institution. Lyduine Collij’s salary is supported by a MSCA postdoctoral fellowship research grant (101108819) and the Alzheimer Association Research Fellowship (grant 23AARF-1029663). Gérard Bischof is funded by the Deutsche Forschungsgemeinschaft (DFG), project 431549029-SFB 1451, and partially by DFG DR 445/9-1. Mark Battle is employed by GE HealthCare. Robin Wolz is employed by IXICO Ltd. Rossella Gismondi is employed by Life Molecular Imaging. Andrew Stephens is employed by Life Molecular Imaging. Zuzana Walker has received research support from GE HealthCare. Philip Scheltens is employed by the EQT Life Sciences team. Agneta Nordberg has received consulting fees from H Lundbeck AB and AVVA Pharmaceuticals and honoraria for a lecture from F. Hoffmann–La Roche. Juan Domingo Gispert has received research support from GE HealthCare, Roche Diagnostics, and F. Hoffmann–La Roche; has received speaker or consulting fees from Roche Diagnostics, Esteve, Philips Nederlands, Biogen, and Life Molecular Imaging; and serves on the Molecular Neuroimaging Advisory Board of Prothena Biosciences. Alexander Drzezga has received research support from Siemens Healthineers, Life Molecular Imaging, GE HealthCare, AVID Radiopharmaceuticals, Sofie, Eisai, Novartis/AAA, and Ariceum Therapeutics and has received speaker fees from and served on honorary or advisory boards for Siemens Healthineers, Sanofi, GE HealthCare, Biogen, Novo Nordisk, Invicro, Novartis/AAA, Bayer Vital, and Lilly. Alexander Drzezga also holds stock in Siemens Healthineers, Lantheus Holding, Structured Therapeutics, and Lilly and a patent for [^18^F]JK-PSMA- 7 (patent number EP3765097A1; date of patent, January 20, 2021). Silvia Morbelli has received speaker honoraria from GE HealthCare, Eli Lilly, and Life Molecular Imaging. Christopher Buckley is employed by GE HealthCare. Valentina Garibotto is supported by the Swiss National Science Foundation (project numbers 320030_185028 and 320030_169876), the Aetas Foundation, the Schmidheiny Foundation, the Velux Foundation, and the Fondation privée des HUG. She has received support for research and speaker fees from Siemens Healthineers, GE HealthCare, Janssen, and Novo Nordisk, all paid to the institution. Gill Farrar is employed by GE HealthCare. Frederik Barkhof is supported by the NIHR biomedical research center at UCLH. He has been a steering committee or Data Safety Monitoring Board member for Biogen, Merck, Eisai, and Prothena; an advisory board member for Combinostics and Scottish Brain Sciences; and a consultant for Roche, Celltrion, Rewind Therapeutics, Merck, and Bracco. Frederik Barkhof has research agreements with ADDI, Merck, Biogen, GE HealthCare, and Roche and is a Cofounder and shareholder of Queen Square Analytics Ltd. No other potential conflict of interest relevant to this article was reported.

## Notes

KEY POINTS **QUESTION:** Does quantification of Aβ PET images support visual assessment of this imaging biomarker in a memory clinic population? **PERTINENT FINDINGS:** In a pan-European cohort across the clinical continuum and enriched for challenging cases in terms of Aβ PET status, we observed a significant increase in reader agreement and confidence after disclosure of the quantitative Aβ PET results across 5 readers with varying levels of experience. **IMPLICATIONS FOR PATIENT CARE:** Achieving high certainty of Aβ PET status at the individual level is key considering the arrival of antiamyloid therapies, which use the quantitative Centiloid metric for trial inclusion and target engagement.
